# Sustained specialized and family treatment in first-episode schizophrenia or related disorders: a 5-year randomized controlled trial

**DOI:** 10.1017/S003329172200099X

**Published:** 2023-07

**Authors:** Lieuwe de Haan, Don Linszen, Luuk Wouters, Koos Zwinderman, Peter Dingemans

**Affiliations:** 1 Department of Psychiatry, Early Psychosis, Amsterdam UMC, Amsterdam; 2 Departement of Epidemiology, Amsterdam UMC, Amsterdam

**Keywords:** schizophrenia, first episode of psychosis, psychotic relapse, social functioning specialist treatment, family treatment, randomized controlled trial

## Abstract

**Background:**

The long-term outcome of first-episode schizophrenia needs improvement. Here, we evaluate the effectiveness of 5 years sustained specialist treatment (ST), ST including Parent groups (ST + P) or treatment as usual (TAU) on psychotic relapse and social functioning.

**Methods:**

A three condition randomized, parallel assigned, single-blind efficacy trial, in which 198 first-episode psychosis (FEP) patients aged 15–28 years were included. The effect on time to first relapse, first relapse rates, mean number of relapses per patient, and time to the improvement of social functioning were analyzed using Cox regression or ANOVA.

**Results:**

We found no significant differences between treatment conditions in the ITT analysis concerning time to first relapse, nor first relapse rate. Mean number of relapses per patient differed at a trend level between ST, ST + P or TAU conditions, respectively: 0.72; 0.62 or 1.02 (*p* = 0.069). No evidence was found for differential effect of treatment conditions on social functioning.

**Conclusion:**

Five years sustained ST of FEP nor addition of parent groups increased time to first relapse or reduced first relapse rate, compared to sustained TAU. Indications for favorable effects of parent groups were found on relapses per patient.

The long-term outcome of first-episode schizophrenia needs to be improved. Follow-up studies have shown, that despite initial remission of psychosis, patients with first-episode schizophrenia tend to experience one or more relapses (Gafoor et al., 2010; Martland, Martland, Cullen, & Bhattacharyya, 2020; van Os & Kapur, 2009; Wiersma, Nienhuis, Slooff, & Giel, 1998). Each relapse is associated with an increased risk of non-remission and functional impairment (Alvarez-Jiménez, Parker, Hetrick, McGorry, & Gleeson, 2011, 2012; Häfner & van der Heiden, 1997; Robinson et al., 1999; Wiersma et al., 1998). Several short term pharmacological and psychosocial treatment studies in early schizophrenia have shown symptom remission, reduced relapse rates and improved social functioning (Crow, MacMillan, Johnson, & Johnstone, 1986; Kahn et al., 2008; Lieberman et al., 2005; Linszen et al., 1996; Marshall & Lockwood, 2004; Penn, Waldheter, Perkins, Mueser, & Lieberman, 2005; Robinson, Woerner, Delman, & Kane, 2005). Randomized controlled family intervention studies in recent onset patients also revealed favorable short-term effects on relapse (Bighelli et al., 2021; Claxton, Onwumere, & Fornells-Ambrojo, 2017; Goldstein, Rodnick, Evans, May, & Steinberg, 1978; Schooler et al., 1997; Zhang, Wang, Li, & Phillips, 1994). Moreover, two randomized clinical trials examining specialist intensive outreach treatment in early psychosis found favorable results after 18 months and 2 years of treatment respectively (Craig et al., 2004; Petersen et al., 2005). However, 5-year follow-up studies revealed a higher relapse rate of up to 82 percent (Bertelsen et al., 2008; Geddes et al., 1994; Scottish Schizophrenia Research Group, 1992). In a first episode trial concerning family intervention we also found a relapse rate of 15% during the first 15 months of treatment (Linszen et al., 1996). However, at 5-year follow-up the relapse rate increased to 52 and 25% of the patients had persistent psychotic symptoms (Lenior, Dingemans, Linszen, de Haan, & Schene, 2001). Puntis et al. (2020) performed a meta-analysis to evaluate whether extended specialized early intervention improved outcome. They found minor uncertain benefits of extended specialized early intervention concerning remission, engagement with services but no clear evidence for less psychiatric hospital admission or days spent in a psychiatric hospital.

Taken together, these findings suggest that specialist or family interventions in the early phase of schizophrenia or related disorders are effective as long as they are active. We need long-term clinical trials covering at least the first 5 years after the first psychotic episode, the so-called critical phase of schizophrenia (Birchwood, Todd, & Jackson, 1998; de Winter et al., 2022; Linszen, Dingemans, & Lenior, 2001; Strauss & Carpenter, 1977). Since treatment continuation and adherence are associated with a therapeutic alliance (Browne et al., 2021), and discontinuity of care may undermine a therapeutic alliance, a specialist treatment (ST) program with continuity of treatment by the same professional during the first 5 years of the disorder may improve symptomatic and functional outcome. Moreover, sustained family intervention may further diminish psychotic relapse and/or improve social functioning during the initial 5 years of schizophrenia or related disorders.

It is important to notice that it is necessary that all patients included in intervention studies were willing to collaborate with clinicians and researchers. This means that results from intervention studies, including current study, are not necessarily generalizable to all patients with recent-onset psychotic disorders.

Here, we report on a three condition randomized, parallel assigned, single-blind efficacy trial with a duration of 5 years in patients with a first-episode of schizophrenia or related disorder and their families, to answer three questions: 1. Does sustained specialist treatment by the same professional (ST) increase time to first relapse, reduce first relapse rate or mean number of relapses compared to sustained treatment as usual (TAU); 2. Does the addition of sustained parent groups to ST (ST + P) contribute to further beneficial effects on time to first relapse, first relapse rate or mean number of relapses?; 3. Are there differential effects of the three treatment conditions on time to improvement of social functioning?

## Methods

### Participants

Patients were eligible if they: (1) were suffering from a first psychotic episode of schizophrenia or a related disorder meeting DSM-IV-R criteria for (American Psychiatric Association, 1994) Patients with substance-induced psychotic disorders were not included; (2) were between 15 and 28 years of age; and (3) were living in contact with (a) parent(s).

Patients were referred for treatment to the early psychosis department of the Academic Medical Centre of the University of Amsterdam. The department has inpatient and outpatient facilities. All mental health services in Amsterdam referred first psychotic episode patients. After a complete description of the study, written informed consent was obtained from all participants. When a participant was younger than 18 years, we also obtained written informed consent from the parents. All consecutively referred patients fulfilling the inclusion criteria and willing to participate were included. Patients were randomized to one of the three treatment conditions. The study was approved by the ethical review board of the AMC.

### Sample size

We based our sample size calculation on our primary objective to detect a true positive effect of one or both of our experimental treatment conditions on time to relapse or relapse rate (ST and ST + P compared with TAU). Based on earlier studies we assumed a 65% relapse rate in the TAU condition (Lenior et al., 2001). We deemed a 25% reduction of relapse as clinical relevant. With testing on a one-sided level of 5%, power was acceptable (89.8%) when we would include 200 patients.

### Assessments

Patients were assessed at baseline, after 1 year, 3 years, and 5 years. Assessments were done by trained and independent raters, blind for the treatment condition.

### Potential prognostic demographic and clinical characteristics

At baseline, the following characteristics were assessed: gender, age of onset of first symptoms, type of onset (acute, insidious), duration of untreated psychosis, premorbid functioning as assessed with the Prognostic Scale (Strauss & Carpenter, 1977) Psychosocial functioning during youth as assessed with the premorbid adjustment scale. The DSM IV diagnosis was based on the Structured Clinical Interview for DSM IV (First, Spitzer, Gibbon, & Williams, 1997) with the use of all available information (interview with the patient and a separate interview with involved family members). Severity of psychopathology was assessed with the Positive and Negative Syndrome Scale (PANSS, Kay, Fiszbein, and Opler, 1987). The intraclass correlation coefficient for the PANSS positive, negative, and general psychopathology subscales were 0.91, 0.84, and 0.76, respectively.

### Outcome: Psychotic relapse, social functioning

We used the Life Chart Schedule (LCS) to assess the timing and number of psychotic relapses, social functioning, medication use and compliance at year 1, 3 and 5 after the start of the trial (Susser et al., 2000). The LCS yields reliable ratings of the long-term course of schizophrenia when assessed by trained raters (Linszen et al., 1994). Trained interviewers not involved in clinical treatment of patients assessed detailed information from respondents (patients and parents) and from clinical records. Respondents were asked to indicate any changes in symptoms, treatment and social functioning since the last assessment.

Psychotic relapse was rated as present when both of the following criteria were met: (1) recurrence or exacerbation of psychotic symptoms with a duration of at least one week and (2) an increase in dosage of prescribed antipsychotic medication (Linszen et al., 1994). We used this definition because we intended to measure clinical relevant psychotic relapse, that could be reliable determined from interviews and LCS data. LCS data with regard to relapse were clinically reviewed by two of the researchers (DL, PD), who were blind for the assigned treatment condition. Data sensitive for the treatment allocation were removed from the LCS by an independent research assistant. We did not observe unblinding. There was initial disagreement about the relapse status of 12 patients (7%). After expert discussion (DL, PD, LH) consensus was reached.

Social functioning was rated in 6-month intervals and determined to be either good or poor: good social functioning was characterized by non-residential housing and participating in education or having a regular job (paid or voluntarily); poor functioning was characterized by residential housing or not participating in education nor having a regular job.

### Treatment

Treatment was given in the following settings: the *in-patient setting* (with a duration of approximately two months) with a highly structured program in which patients and parents participated in therapeutic and psycho-educational programs and an *outpatient setting* (with a duration of approximately 60 months). Patients who did not agree to be admitted started with outpatient treatment. Both inpatient and outpatient programs have been described in detail elsewhere (Linszen et al., 1996). Pharmacological treatment was intended to achieve remission of psychotic symptoms without substantial side effects. Consequently, low dosages of antipsychotic medication were used. Patients, and parents, were offered emotional support and educated about the nature and treatment of the illness. Intensive and sustained support was provided to improve social functioning.

The three outpatient treatment conditions were characterized as follows:

ST was given by the professional staff of the early psychosis department of the AMC. Continuity of care in treatment and in professional caregiver was provided for 5 years. During the complete study period the treatment team was formed by two psychiatrists and three psychiatric nurses and two-family therapists. There were no personnel changes throughout the study period. Treatment targets were described in manuals and included relapse prevention and prevention of psychotic symptom exacerbation through recognition of prodromal symptoms, improving adequate coping, improving medication adherence, reducing substance use, preventing drop-out and suicide, improving selfconfidence and mood and supporting participation in work and/or education.

In the ST + P condition patients were treated as in the ST condition with the addition of parent groups. The parent-groups intervention was based on the family management approach as developed by Goldstein and collaborators (Anderson, Reiss, & Hogarty, 1986; Goldstein et al., 1978; Falloon et al., 1982). We used an adaptation developed for parents of first psychotic episode patients, which we described in an earlier study (Linszen et al., 1996). The first sessions focused on crisis intervention, followed by psycho-education and training in problem solving. Approximately thirty parent group therapy sessions, with 6–8 parents each, were held over a 60-month period. Each parent group was led by two experienced family therapists. It is important to note that we did not offer CBT nor family intervention involving the patient.

TAU was provided during 5 years by local mental health care professionals situated nearby the domicile of patients. Most referred patients who were randomized to TAU resumed, after inpatient treatment, their contact with the professional caregiver who initially referred them to our department. TAU had comparable treatment targets as ST (it was sustained and dedicated to prevent psychotic relapse and to improve social functioning). However TAU was not provided by treatment staff specialized in early psychosis intervention. Professionals in the TAU condition intended to continue antipsychotic medication according to the former guidelines and held contact with family members without offering specific family treatment. Treatment in the TAU condition was not controlled and varied. Although some patients in the TAU condition received cognitive behavioral therapy, this occurred seldom.

### Antipsychotic medication adherence

Antipsychotic medication adherence was assessed as a crucial risk factor for psychotic relapse.

An independent research assistant, blind to the condition assignment, rated adherence to antipsychotic medication throughout the study period as: poor (0–24% of prescribed medication taken), irregular (25–49%), or good (regular intake: 50–74% or depot medication).

### Statistical analysis

Time to first psychotic relapse was analyzed with survival analyses. First psychotic relapse data were estimated using life-table methods (Kaplan Meier), with 95% confidence intervals (CIs) estimated by the arcsine square root approximation. Treatment effects were estimated by Cox regressions and were done both with and without possible confounding variables. Log-Minus-Log survival plots were used to determine if baseline hazard functions were proportional across conditions.

First relapse rate and mean number of relapses per patient were compared between conditions with ANOVA.

Several patients changed from the treatment condition they were randomly assigned to. Therefore, we exploratively carried out additional analyses in which patients were analyzed in their actually realized treatment condition (ART). Since the comparison of the treatments in the ART analysis was no longer based on randomized allocation and therefore could be confounded by patient characteristics, we performed three analyses to assess the treatment effects on relapse risk controlled for potential prognostic factors. First, we performed multiple Cox regression analysis of the treatment effects corrected for gender, ethnicity, education, DUP, early onset, type of onset, prognostic and premorbid scores, PANSS total, negative and positive symptom scores at baseline, substance abuse, adherence and insight. Second, we compared treatment effects within strata of patients with similar risk of relapse according to the same set of confounders. Third, we estimated for all individuals the propensity to relapse for the three treatments according to the same set of confounders using (nominal) logistic regression. The treatment effects were subsequently compared using Cox regression using inverse probability weighing using the propensity probabilities of the nominal regression analyses.

Time to improvement of social functioning was also analyzed with ITT and ART Cox proportional hazards analyses.

Trial registration number: ClinicalTrial.gov identifier NCT01936220.

## Results

### Characteristics of the study sample

**Fig. 1.**
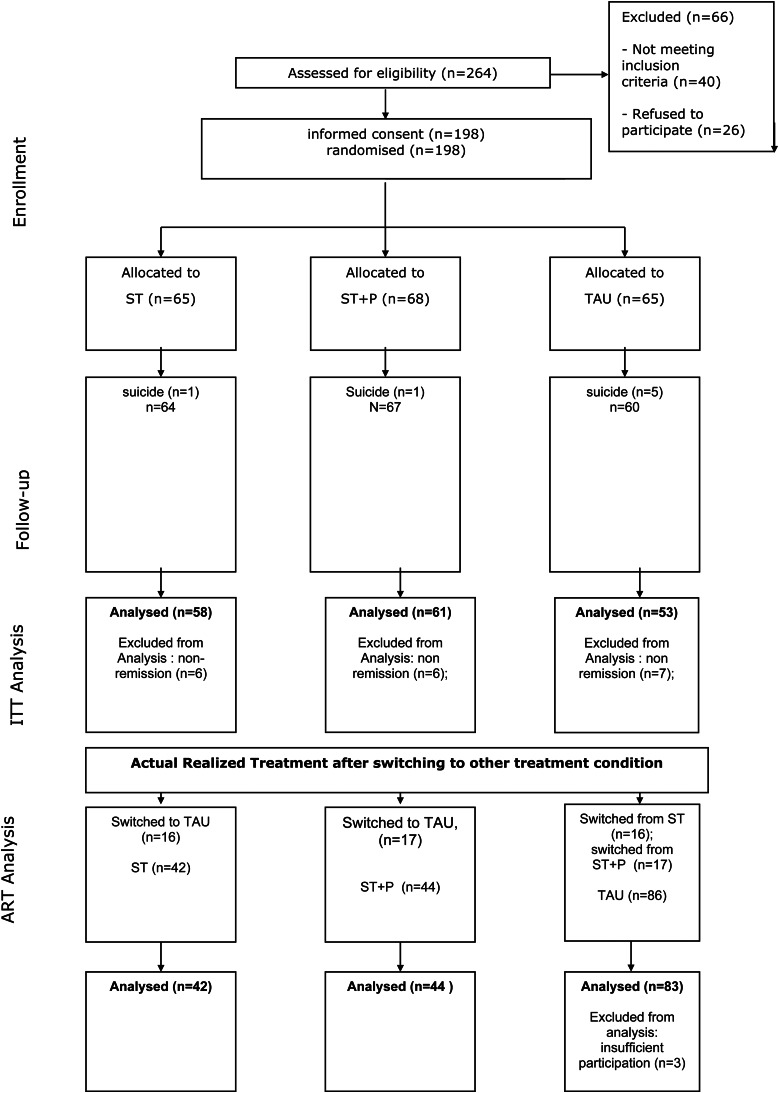
Consort diagram.

Two hundred and sixty-four referred patients were assessed for eligibility. One hundred and ninety-eight patients met all inclusion criteria and gave written informed consent. One hundred and seventy-six were admitted to the inpatient unit, and 22 (11%) preferred to start the study as outpatients. DSM-IV diagnoses of the included patients were schizophrenia (*n* = 108, 55%), schizoaffective disorder (*n* = 42, 21%), schizophreniform disorder (*n* = 26, 13%) or other psychotic disorders (*n* = 22, 11%). Before the start of the outpatient phase 65 patients were randomly assigned to the ST condition, 68 patients to ST + P condition and 65 patients to the TAU condition. Complete data on relapse and social functioning were obtained from 192 patients (97%) in year 1, 182 patients (87%) in year 3 and 152 patients (77%) in year 5. For all patients included in the ITT we obtained data on psychotic relapse during 5 years (Fig. 1).

Table 1 shows the baseline demographic and clinical characteristics of all 198 patients.

**Table 1.** Demographic and clinical characteristics at baseline of participants with tests on differences between both ITT and ART conditions

	All patients *N* = 198	ITT TAU *N* = 53	ITT ST *N* = 58	ITT ST + P *N* = 61	ITT *N* = 172 *p*^a^	ART TAU *N* = 83	ART ST *N* = 42	ART ST + P *N* = 44	ART *N* = 169 *p*^a^
Age at referral (mean, s.d.)	21.3 (2.9)	21.3 (3.3)	21.7 (2.9)	21.2 (2.6)	0.566	21.4 (3.1)	21.5 (2.9)	21.3 (2.6)	0.978
Male (*n*;%)	162 (82%)	41 (77%)	48 (83%)	52 (84%)	0.639	63 (76%)	36 (86%)	39 (87%)	0.227
Caucasian (*n*;%)	137 (71%)	37 (70%)	46 (79%)	41 (66%)	0.260	60 (72%)	30 (71%)	33 (73%)	0.980
Secondary school finished (*n*;%)	105 (53%)	29 (55%)	30 (52%)	38 (61%)	0.557	44 (53%)	22 (52%)	28 (62%)	0.551
Strauss Carpenter prognostic scale (mean, s.d.)	39.7 (10.8)	42.5 (10.3)	38.2 (10.2)	40.7 (11.8)	0.111	41.2 (10.0)	38.3 (10.5)	40.9 (12.8)	0.357
Premorbid adjustments scale (mean, s.d.)	14.5 (6.1)	15.4 (6.4)	14.5 (5.9)	14.2 (5.8)	0.541	15.0 (6.1)	14.7 (6.1)	13.6 (5.8)	0.442
Duration untreated psychosis 1 year or longer (*n*; %)	59 (30%)	13 (25%)	18 (31%)	19 (31%)	0.700	22 (27%)	12 (29%)	15 (33%)	0.717
Type of onset (*n*;%)									
Acute	23 (12%)	6 (11%)	5 ( 9 %)	11 (18%)	0.137	11 (13%)	3 ( 7%)	8 (18%)	0.227
Subacute	70 (35%)	22 (42%)	15 (26%)	23 (37%)		29 (35%)	11 (26%)	18 (41%)	
Insidious	104 (53%)	25 (47%)	38 (65%)	27 (45%)		43 (52%)	28 (67%)	18 (41%)	
Onset before age 18 (*n*;%)	60 (30%)	16 (30%)	13 (22%)	20 (32%)	0.458	22 (27%)	12 (29%)	14 (31%)	0.857
PANSS positive symptoms score (mean s.d.)	20.7 (7.6)	20.6 (7.5)	19.7 (7.8)	21.4 (7.2)	0.494	21.0 (7.7)	19.0 (7.4)	21.2 (7.1)	0.282
PANSS negative symptoms score (mean s.d.)	21.5 (7.2)	21.7 (6.0)	21.4 (6.5)	21.2 (7.9)	0.946	22.5 (6.6)	21.4 (6.6)	20.1 (7.2)	0.156
PANSS general psychopathology symptoms score (mean s.d.)	40.7 (10.7)	39.4 (8.6)	40.7 (12.0)	40.2 (10.6)	0.823	41.3 (9.5)	39.0 (12.1)	39.6 (10.7)	0.444
Voluntary admission (*n*; %)	181 (94%)	51 (96%)	55 (95%)	59 (95%)	0.936	78 (94%)	40 (95%)	44 (98%)	0.625
Non-remission at start outpatient phase (*n*; %)	19 (10%)	7 (12%)	6 (9%)	6 (9%)	0.853^b^	11 (12%)	3 ( 7%)	4 ( 8%)	0.593^b^

a *p* = probability in significance test, i.e. analysis of variance or χ^2^ according to level of measurement.

b All variables (except the variable ‘non-remission at start outpatient phase’) were tested twice, including and excluding those who where in non-remission chronic at discharge. Not one test yielded significant differences between conditions. Only tests excluding patients with non-remission are reported, except the test for non-remission itself for obvious reasons.

All variables in Table 1 were tested for significant differences between the 152 patients who had fully participated for 5 years in the study and those who did not (*n* = 46). Within the first 3 years of the study 7 out of these 46 patients were lost due to suicide (5 in the TAU condition). Within the full participating group Caucasian ethnicity was significantly more predominant (83% *v.* 69%; *p* = 0.029) and also initial voluntary admission (81% *v.* 55%; *p* = 0.038).

### Remission, suicide and relapse

Patients who did not achieve remission (remission was defined as psychotic symptoms are absent or do not have any influence on functioning) (*n* = 19, 10%) or patients who died by suicide (*n* = 7, 4%) were not included in analyses concerning relapse outcome, leaving 172 patients suitable for the ITT survival-analysis. No relapse during 5 year was observed in 91 patients (53%) and 81 patients (47%) relapsed once or more.

### Relapse: ITT analysis

We found no significant effect of treatment condition on time to relapse (*p* ⩽ 0.74 without correction and *p* ⩽ 0.84 with correction for confounding) nor first relapse rate. No significant differences between conditions for potential prognostic variables (see Table 1.) nor violations of proportionality were found. Mean number of cumulative relapses per patient was different at a trend level between treatment conditions: 0.72 in the ST condition, 0.62 in the ST + P condition and 1.02 in the TAU condition (*p* = 0.069). In the ST condition we observed 28 first relapses, 15 s relapses, 4 third relapses, leading to a total of 47 relapses. In the ST + P condition we observed 25 first relapses, 10 s relapses, 5 third relapses, leading to a total of 40 relapses. In de TAU condition we observed 28 first relapses, 26 s relapses, 12 third relapses, leading to a total of 66 relapses.

### Relapse: ART analysis

Sixteen of patients allocated to the ST condition and 17 of the patients allocated tot the ST + P condition switched to the TAU treatment condition, but none changed from the TAU condition. Patients switched within a year after the start of the study due either to inconvenient traveling distance to the university hospital (60%), availability of treatment in their neighborhood (24%), or unknown reasons (16%). One pair of parents left the parent group condition due to language problems. We excluded two patients from the ART analysis who dropped out of treatment during the first year. Switches of treatment conditions occurred before relapse. Thus 170 patients remained for the Actual Realized Treatment (ART) analysis with 42 patients in the ST condition, 45 in the ST + P condition and 83 in the TAU condition. Potential prognostic variables were tested for significant differences between ART conditions and between those who did (*N* = 33) and did not switch (*N* = 137). No significant differences were found (see Table 1).

Cox regressions, both with and without correction for possible confounders using the same confounders as in the ITT analysis were carried out. In the ART analyses ST + P showed a significant effect in reduction of relapse compared with TAU without correction for confounders: hazard ratio (HR) of 0.44 (95% CI 0.24–0.83, *p* ⩽ 0.01) and with correction for confounders: HR 0.44 (95% CI 0.23–0.86, *p* ⩽ 0.02).; ST *v.* TAU showed no significant effect without correcting for confounders: HR of 0.89 (95% CI 0.54–1.49, *p* ⩽ 0.67 nor with correction: HR 0.88 (95% CI 0.51–1.49, *p* ⩽ 0.63).

Stratified for relapse risk according to the list of confounders, the results were essentially the same: HR of ST + P *v.* TAU was 0.5 (95% CI 0.3–1.0) and the HR of ST *v.* TAU was 1.1 (95% CI 0.7–1.8). After inverse probability weighing the results were also comparable: HR of ST + P was 0.4 (95% CI 0.2–0.8) and the HR of ST *v.* TAU was 0.9 (95% CI 0.5–1.6). The first relapse rate during a period of 5 year's treatment was 53% (95% CI 38–67%) in the ST condition, 30% (95% CI 18–45%) in the ST + P condition and 56% (95% CI 45–67%) in the TAU condition.

### Social functioning

At the start of the outpatient phase 109 patients had poor social functioning, of whom 71 showed improvement (65%) and 38 patients did not change (35%). ITT and ART Cox regression analyses concerning time to social improvement for those patients who started with poor social functioning were repeated four times: including and excluding chronic patients, with or without all confounders that were used for the relapse analyses. None showed significance in either ITT nor ART analyses. χ^2^ tests also did not show any significant relation between ITT or ART conditions and categories of psychosocial functioning, neither with nor without non-remitting patients included.

Importantly, 44 patients (13 in the ST condition, 12 in the ST + P condition and, 19 in the TAU condition) of the 89 patients who started with good social functioning at the start of the outpatient treatment phase were rated with poor social functioning at 5 year outcome.

## Discussion

To the best of our knowledge, this is the first randomized controlled trial covering 5 years of the initial phase of schizophrenia or related psychotic disorders. We tested whether sustained specialist intervention or adding parent groups to sustained specialist intervention during the first 5 years of the disorder reduced time to first relapse, first relapse rate or mean number of relapses per patient compared to TAU. We found no significant difference in effect of conditions on time to first relapse nor on first relapse rate. However, we observed trend-level differences in mean number of relapses per patient in favor of the specialist intervention plus parent groups condition.

Three observations are important in interpreting these results. First, all treatment conditions were associated with a relatively low relapse rate of around 50% in 5 years. This finding contrasts the grim results of earlier 5 year follow up studies showing that the beneficial effects of first episode intervention programs with a duration up to 2 years on psychotic relapse disappeared at 5-year follow-up with relapse percentages varying from 70 to 82% at year 5 (Bertelsen et al., 2008; Geddes et al., 1994; Hegarty, Baldessarini, Tohen, Waternaux, & Oepen, 1994). Therefore, the results of our study may indicate that both continuity of ST as well as continuity of TAU prevent relapse compared to shorter interventions. Continuity of ST in the same institution and by the same staff during the in- and outpatient phase over a longer period of time appeared not to be crucial in reducing first relapse when compared to TAU offered by non-specialized mental health care professionals. It may be noteworthy that patients assigned to the sustained TAU condition were mostly treated by professionals who *referred* them to our specialist center, possibly illustrating the importance of continuity of care by caregiver. Second, despite a focus on recognition of suicidality, suicide risk remains high (4%), in the 5 years of the trial. Most suicides occurred in the TAU condition, however, our study is not powered to detect differences in suicide rate. This finding highlights the need for better detection and intervention strategies for suicidality, especially in the initial phase of schizophrenia. Third, continuity of ST combined with parent-groups as actually realized did have a significant beneficial effect: only thirty percent of first-episode patients whose parents participated in parent groups relapsed during 5-year treatment. Since this effect may be confounded by patient characteristics and since we found only a trend level difference in mean relapse rate favoring the ST and ST + P condition in our ITT analysis abovementioned favorable effects in the ST + P ART should be interpreted very cautiously. Selection bias may have driven this effect and controlling for possible confounders does not guarantee that the outcome of the ART analysis is driven by the intervention and not by patient or family factors.

We found no significant difference in improvement in social functioning between treatment conditions. Although social functioning improved in most patients in a 5-year period, irrespective of treatment condition, functional impairment developed in a substantial minority of our patients. This result is not in line with findings from a recent meta-analysis that found that family intervention improved functioning of patients (Claxton et al., 2017). Apparently, none of the sustained intervention conditions was more effective concerning social functioning in this subgroup. Improving social functioning probably needs interventions like individual placement and support. An explanation for our failure to find an effect on social functioning may be that we used a binary outcome measure of social functioning that lacks sensitivity. Although social functioning is both a multidimensional concept and a dimensional measure, we decided to use a binary measure because we wanted to assess differences in time to improvement of social functioning, and therefore we needed a clear-cut assessment of improvement. Moreover, our study was not powered to detect more subtle differences in subdomains of social outcome between conditions.

### Strengths and limitations

Our study has several strengths. First, we were able to include a relatively large sample of consecutively referred patients with schizophrenia or related disorders with a first psychotic episode. Second, we performed a randomized comparison of sustained interventions during the first 5 years of the disorder. Third, the dropout rate was relatively low.

However, we need also to acknowledge several limitations. First, despite a good collaboration with all mental health services in Amsterdam we may have missed potential participants. Second, we were not able to include patients who refused any treatment or who were in such a state that treatment in a closed ward or a forensic institute was needed. Therefore, the generalization of our findings to all patients with a first psychotic episode of schizophrenia or related disorders may not be justified. However, we are confident that our findings apply for the majority of patients who are willing to collaborate with professional caregivers. Third, it is important to acknowledge that since our primary outcome measure was psychotic relapse it was necessary that patients would achieve remission to be included in the main analyses. Without achieving remission, it is not possible to relapse. This made that we focused on those patients with a more favorable prognosis. However, it is worth mentioning that there were no significant differences in the proportion of patients not achieving remission between the conditions.

Fourth, although most patients showed improved social functioning, the examined interventions may have been too weak to substantially improve functioning. Possibly a focus on relapse prevention is not easy compatible with a focus on improving social functioning. Fifth, substantial switching from allocated specialist intervention to TAU occurred. This may have influenced outcome in the ITT analyses. Sixth, although psychotic relapse and social functioning are relevant outcome measures, current study lacks other important outcome measures, partly independent of symptomatic outcome, like personal recovery or other patient-reported outcome measures (Leendertse et al., 2021; Van Eck, Burger, Vellinga, Schirmbeck, & de Haan, 2018). Finally, our focus on psychotic relapse and social functioning made that we also did not assess other important outcome measures such as mood and anxiety. Although, depressive symptoms are most strongly associated with quality of life (Van Eck et al., 2018) and personal recovery and although emotional disturbance may contribute to the vulnerability for psychotic relapse. However, this does not mean that interventions aimed at improving emotional disturbances were lacking in the three treatment conditions.

In conclusion, this first randomized trial examining sustained specialist and family intervention covering 5 years of the initial phase of schizophrenia or related disorders revealed no significant difference in psychotic relapse between conditions in the intention to treat analyses. All treatment conditions were associated with a relatively low relapse rate. Possibly, sustained treatment of first-episode patients for at least 5 years offers the promise of reducing psychotic relapse. No differences between conditions concerning improving or preventing functional impairment were found. However, actual realized participation of parents in family treatment was associated with a lower first relapse rate. Therefore, sustained participation in the treatment of parents during the initial phase of psychosis may be associated with an improved outcome.

## Previous presentation

Congress of the Netherlands Association for Psychiatrists 2 April 2018 Maastricht.
